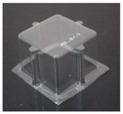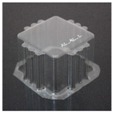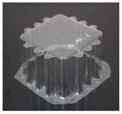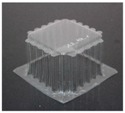# Evaluation of Reliefs’ Properties on Design of Thermoformed Packaging Using Fused Deposition Modelling Moulds

**DOI:** 10.3390/ma12030478

**Published:** 2019-02-04

**Authors:** Lucía Rodríguez-Parada, Pedro F. Mayuet, Antonio J. Gámez

**Affiliations:** Department of Mechanical Engineering & Industrial Design, Faculty of Engineering, University of Cadiz, Av. Universidad de Cádiz 10, E-11519 Puerto Real-Cadiz, Spain; pedro.mayuet@uca.es (P.F.M.); antoniojuan.gamez@uca.es (A.J.G.)

**Keywords:** packaging design, product design, mechanical properties, thermoforming, tensile test, 3D printing, simulation, technology

## Abstract

The increased consumption of food requiring thermoformed packaging implies that the packaging industry demands customized solutions in terms of shapes and sizes to make each packaging unique. In particular, food industry increasingly requires more transparent packaging, with greater clarity and a better presentation of the product they contain. However, in turn, the differentiation of packaging is sought through its geometry and quality, as well as the arrangement of food inside the packaging. In addition, these types of packaging usually include ribs in the walls to improve their physical properties. However, these ribs also affect the final aesthetics of the product. In accordance with this, this research study analyses the mechanical properties of different relief geometries that can affect not only their aesthetics but also their strength. For this purpose, tensile and compression tests were carried out using thermoformed PET sheets. The results provide comparative data on the reliefs studied and show that there are differences in the mechanical properties according to shape, size and disposition in the package.

## 1. Introduction

Thermoformed food packaging, usually called rigid or semi-rigid containers, have as main functions protection, containment, preservation and distribution [1,2]. Different types of thermoformed packaging can be observed in the market according to the specific needs of the food. These include heat-sealable containers for processed or semi-processed products [3,4]. Packaging usually applied to fresh products, such as fruit or vegetables [5], all of which are therefore often used for products with a short life cycle and with the aim of protecting and making food more functional [6]. For this reason, during the development of these containers, the aim is to reduce the amount of material used to increase sustainability by reducing the large amount of waste generated [7]; and to optimise production costs while maintaining their functional properties [5].

Although distribution is one of their main functions [8,9], most of these packages reach the consumer, especially because of the new trend of food on the move [10]. For this reason, the design and finish of these packages must also be taken into consideration. Thus, the ergonomic and functional aspects of the packaging must be aimed at adapting the product to the consumer’s needs for use and protection of the food it contains [2].

Throughout the short life cycle of freshly packaged food there are very different situations to which the packaging is exposed to external aggressions. This is linked to the sustainability of both the product inside and the cost of material involved. Thus, food packaging can delay and protect food from physical, chemical and biological spoilage, that is, packaging can extend shelf life and ensure product quality [7] and therefore can be understood as a sustainability system for food. Light, humidity, microorganisms, shocks and other mechanical forces are examples of some of the external agents that can adversely affect food [2]. In general, both the handling of the package, its transport or its stacking on the supermarket shelf are considered external aggressions to the food contained inside before consumption [5]. Therefore, elastic deformation of the packaging may be required to preserve its content.

Specifically, the weight of the food generates tensile stresses in the walls. One of the situations where this occurs is when the container is suspended during transport or handling. Also, during transport the containers are normally stacked and this means that the container must also withstand the stacking weight. These mechanical forces cause a compressive stress on the container walls. Also, in situations where a user, whether a consumer or not, handles the packaging, a functional situation occurs where the packaging must protect the food [11].

According to these approaches, thermoformed containers usually incorporate patterns on their walls in the form of reliefs, which are used to give them some mechanical properties, without the need to increase the thickness of the plastic sheet used during the manufacturing of the packaging. In addition, reliefs can attribute a different geometric aspect by creating a semantic value on the product. However, the containers currently used in the market usually incorporate reliefs in the form of vertical ribs or columns, generally with square geometry.

A related point to consider is the package visual appearance, which influences the decision of consumption due to its symbiotic or simply aesthetic qualities [12,13]. In these circumstances, the container may be a key factor in the consumer decision-making, allowing inferences to be drawn about the product, like attributes or taste. Therefore, the package can influence subsequent experiences in the product so much so that researchers study its influence concerning materials, shapes and sizes [3,13,14,15,16,17], although only a few studies focus on the tactile sensation that a container causes. This is a new trend in packaging, such as in cosmetics or drinks [18]. As a designed texture or relief using differentiating factors can attract consumers as much as shape or colour, designing custom geometries is of utmost interest, although it may alter the functional properties of the package.

Given the above conditions, the influence of relief geometries on the mechanical properties of thermoformed sheet is studied and analysed in this work. By means of this method, the properties of the reliefs of the packaging can be evaluated in order to guarantee sustainability and also to reduce the expense of plastic material. Furthermore, the improvement of the mechanical properties of the containers also contributes to increase the useful life of the food. It should be also stressed that, conventionally, reliefs are not specifically evaluated until the physical package is tested. This means that design and development times can be increased by design modifications.

For this purpose, Fused Deposition Modelling (FDM), an Additive Manufacturing (AM) technology, is proposed for making economic moulds that allow the thermoforming of test specimens. AM refers as a general term to manufacturing technologies that build up a product layer by layer [19]. Since the 1980´s, AM first started unsteadily and then moved from laboratory to industrial practice. New applications are constantly announced and they have been developed over time [20]. Thus, the applications of this technology have been increasing. Despite the fact that AM processes, such as Stereolithography (SLA), FDM or Selective Laser Sintering (SLS) were initially created for the purpose of generating rapid prototypes, it is currently sought to use them to create final products as well [21,22].

In the context of thermoformed products, moulds from additive manufacturing are being created nowadays [23]. Specifically, the applications focus on the study of this technology in the field of design and development processes, in order to create moulds for short series [24]. To do this, additive manufacturing is used to validate proposals as well as for research support, as it allows to generate moulds in a fast and economic way [25,26].

In the field of thermoforming, Laser Sintering is widely used. In this research, in contrast, the application of the FDM technology is considered since it is an affordable technology and accessible to any design team [27]. In effect, the use of FDM offers the possibility of creating moulds of high quality, though it may also be slow and costly [28]. Thus, printers provide the possibility of generating moulds in order to create thermoforming prototypes in a rapid way, resulting in shortened deadlines and reduction of errors.

This work presents an experimental procedure that provides a novel scheme to evaluate the relief patterns designed on food packages with a double objective: to improve the mechanical resistance without increasing the quantity of raw material and to build more efficient products. Moreover, this evaluation technique, can be used with thermoplastic materials like Polyethylenne Terephtalate (PET), due to their predominance in disposable containers but can be extended to new biodegradable materials such as Bio Polyethylenne Terephtalate (Bio-PET) and Polylactic Acid (PLA). In addition, its application could be extended to other materials such as thin cardboard sheets.

In short, additive manufacturing allows new applications and procedures to emerge in the field of engineering, as is the case with the work proposed here.

## 2. Construction of Test Specimens

In this work, a study of the mechanical behaviour of reliefs has been carried out. Initially, the reliefs were evaluated as a unit, performing tensile tests with the aim of comparatively analyse the existing differences between geometries and relief sizes. After that, the influence of the position and number of the reliefs on the faces of a container, used as a test tube, was studied by performing compression tests.

**Figure 1:**
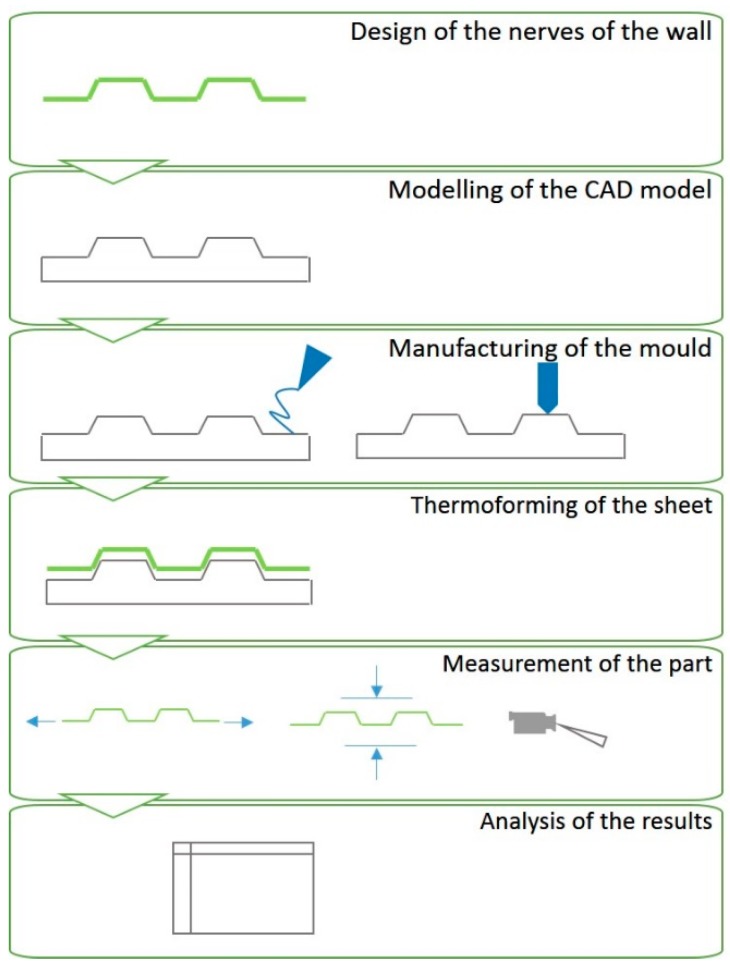
Methodological procedure.

The overall procedure for the manufacture and preparation of the specimens consists of several phases, as seen in Figure 1. Specifically, the generation of the specimen and mould in Computer Aided Design (CAD) was done by using the Solidworks^®^ software (2016 version, Dassault Systèmes SE, Velizy-Villacoublay, France) and the 3D printing of the specimen was done with the Simplify3D^®^ software (Cincinnati, OH, USA) for the generation of the G-code. For the manufacture of the mould of the specimen printed in FDM, a 3D printer machine Witbox^®^ and PLA filament with a diameter of 1.75 mm have been used. The machine used for thermoforming of the sheet was the table-top thermoforming machine Formech 450DT. Finally, Minitab^®^ software (18 version, Minitab Inc., State College, PA, USA) was used for the treatment of the statistical data.

### 2.1. Procedure for the Construction and Preparation of Tensile Specimens

**Figure 2:**
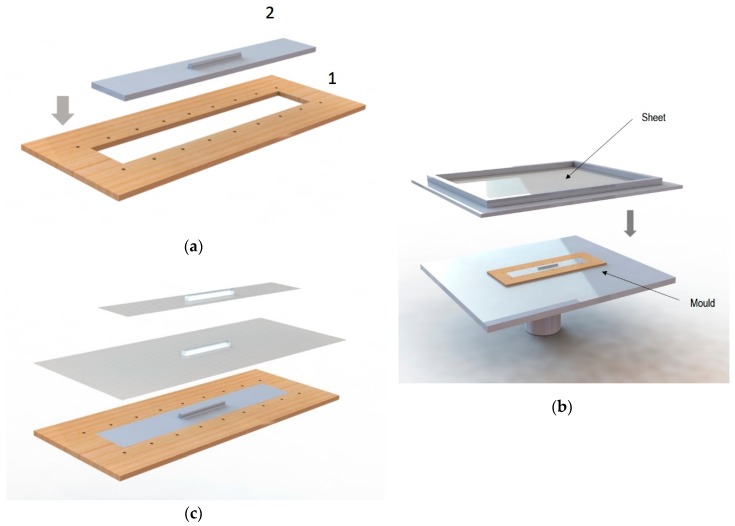
Thermoforming of specimens for tensile tests: (**a**) Hybrid mould; (**b**) Thermoforming of the sheet with hybrid mould to obtain the thermoformed sheet specimen; (**c**) Procedure for obtaining the thermoformed specimen.

In the case of the generation of tensile specimens, to ensure the flatness on the surface, a two parts hybrid mould was designed, Figure 2a. One part was common to all the specimens (labelled 1 in Figure 2a), consisting of a wooden profile with perforations along the perimeter to ensure that the sheet was fixed along the entire surface during the thermoforming. This profile also served as a cutting template to remove the excess of material to ensure repeatability during specimen creation. The second part corresponded to the mould of the specific specimen obtained by FDM (labelled 2 in Figure 2a) and it was fitted into the wooden profile to generate a mould with a flat surface, Figure 2b. This ensured that there were no roundings along the perimeter of the base of the specimen. 

Then, Figure 2c shows the procedure for obtaining the final specimen. Once the specimen was thermoformed, the PET sheet was cut. The wooden profile was used to mark the cutting perimeter over the thermoformed sheet. To make the cut, a sheet shear has been used, according to [29].

### 2.2. Procedure for the Construction and Preparation of Specimens for Compression Tests

**Figure 3:**
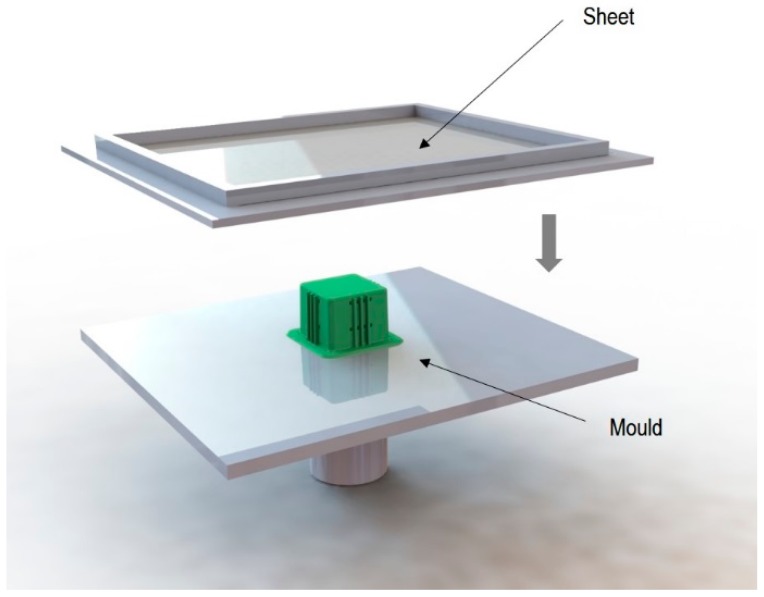
Thermoforming process of specimens for compression tests.

The specimens used for compression tests had a square shape. A mould made entirely of FDM, Figure 3, was used for their construction, as it had flat sides and this way it could be compared with the traction experiments.

**Figure 4:**
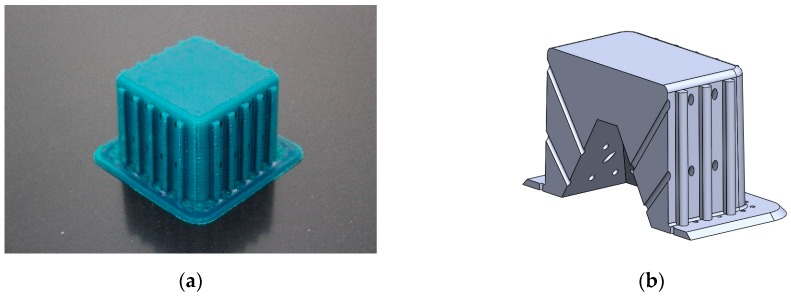
Example images of the mould used for thermoforming compression specimens: (**a**) image of the mould, (**b**) vacuum channels included inside the mould.

The generated mould, Figure 4a, contained a draft angle and a base with a 45° chamfer to avoid the appearance of defects on the thermoformed sheet, in accordance with [30]. It also contained vacuum channels to ensure proper attachment of the PET sheet to the mould. The channels have been made at three heights along the walls of the mould with an angle of 45°, Figure 4b.

Once the thermoforming process had been carried out, Figure 3, the excess of material has been eliminated using the Iberolaser IL-1390 laser cutting machine. This cutting procedure was precise and left no relevant defects on the cutting surface.

## 3. Materials and Methods

### 3.1. Tested Material

The material used in this test was a three-layer PET laminate roll with a recycled PET sheet inside and the thickness used was 180 micrometres. This material is commonly used for food packaging in industrial applications. The material had undergone an extrusion manufacturing process. The properties provided by the supplier are detailed in Table 1, according to UNE-EN-ISO 527-3 [31] and DIN 53479-B [32].

### 3.2. Tensile Test Method

The main objective of these tensile tests was to deepen the knowledge of the behaviour of the different geometries in relation to the dimensions of the sheet protrusion. In addition, for the correct evaluation of the reliefs, a morphological study of the thermoformed geometries was performed. For this reason, the reliefs were included in a unitary way on each specimen. 

#### 3.2.1. Morphological Study

**Figure 5:**
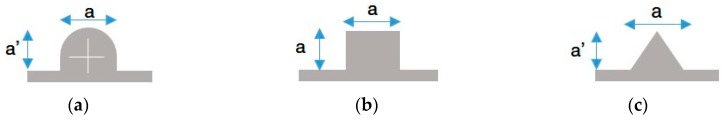
Geometries of the studied reliefs: (**a**) semi-circular, A; (**b**) square, B.; (**c**) triangular, C.

A total of 9 different types of test tubes, based on three different reliefs, were studied. On one hand, three basic geometries were analysed: semi-circular (A), square (B) and triangular (C). These geometries were transferred to the PET sheet in a straight line. On the other hand, three sizes or scale relationships were studied: a, a/2 and 2a where a was 3 mm in the designs, Figure 5. That particular value was chosen after analysing the reliefs of different commercial packages.

Obviously, the section, S, of these three geometries is different, directly affecting the mechanical properties, Equation (1).

(1) $$\sigma =F_{y}/S,$$

where *F_y_* corresponds to the axial force applied to the specimen, either in tensile or compression tests.

**Figure 6:**
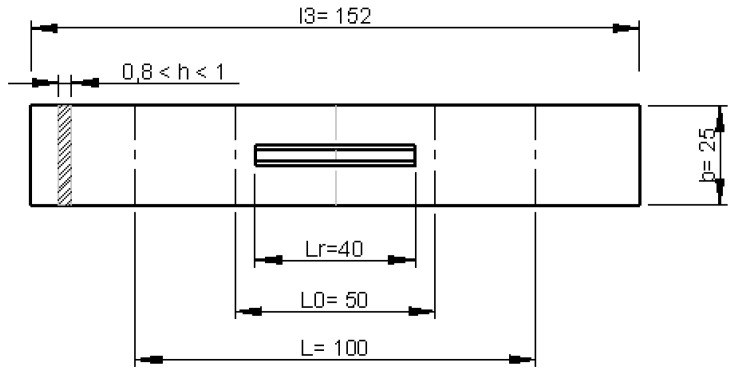
Dimensions of specimen according to standard [31].

Sections were easily calculated as the chosen reliefs were simple geometrical shapes. Relief type A corresponded to a semicircle attached to a rectangle, Figure 6a. Relief B was chosen to be a square, Figure 6b, while relief C was designed as a triangle, Figure 6c. Their respective sections are:

(2) $$S_{A}=\left(\pi *\frac{a^{2}}{8}\right)+a*\left(a^{\prime }-\frac{a}{2}\right),$$

(3) $$S_{B}=a^{2},$$

(4) $$S_{C}=\left(a*a^{\prime }\right)/2$$

#### 3.2.2. Mechanical Characterization Study

**Figure 7:**
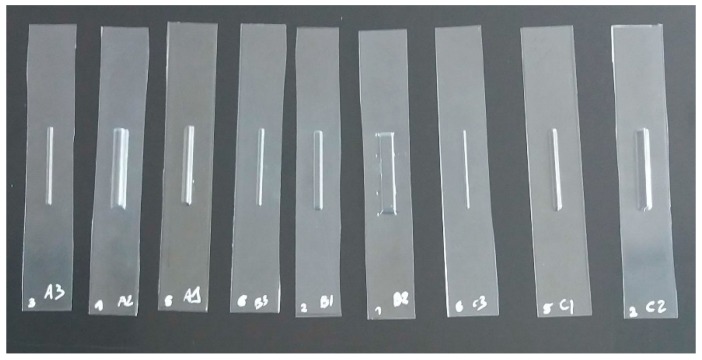
Thermoformed specimens.

In accordance with standard UNE-527-3 [31], Figure 6, the designed dimensions used for a’ and l3 in the test specimen corresponded to 25 mm and 152 mm, respectively. All the other relevant dimensions are depicted in Figure 7. 

The guidelines established in References [31] and [33] had been taken as a reference for carrying out the tensile tests. Thus, a total of 5 specimens per geometry were studied.

**Figure 8:**
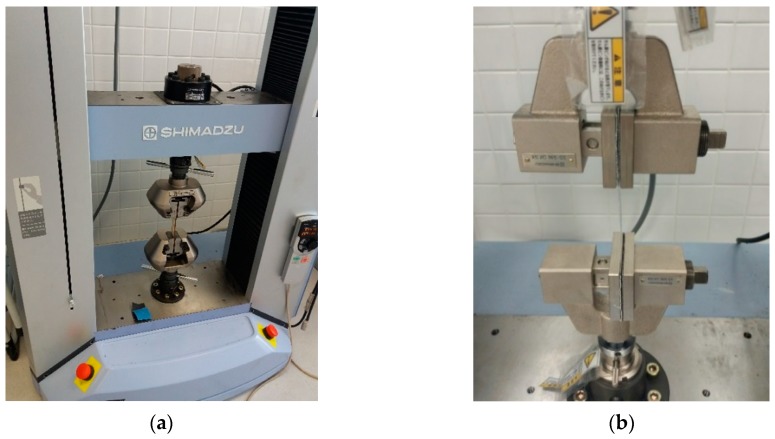
Testing machine with clamps for tensile tests: (**a**) testing machine for the experimental (**b**) clamps with specimen in the middle.

The machine used for mechanical testing was the equipment Shimadzu, model AG-X, with a load cell of 50 KN, Figure 8a. All test parameters were managed with the help of the universal test software Trapezium^®^ for Windows^®^. For the development of the tests, plastic-specific jaws were used, Figure 8b. 

After the laboratory tests, from which experimental data were extracted, simulations of the tensile tests using the Finite Elements Method (FEM) were carried out with the software Solidworks^®^ and Hyperworks^®^ Radios for geometric modelling and dynamic simulation, respectively.

Due to the fact that the range of displacement increases with the test time, the study time was defined at 6 s.

### 3.3. Method for Compression Tests

The objective of the compression tests, with a structure similar to that used for tensile tests, is to deepen the knowledge of the behaviour of the reliefs included on the walls of the packaging. The main idea of this study is to evaluate the influence of the number of reliefs on the packaging and the distance between them.

1, 3 and 5 reliefs had been inserted on each face in order to study their influence on the mechanical properties. In this case, the study was restricted to the study of type A geometry, Figure 5. All the reliefs were placed symmetrically from the centre of each face. 

#### 3.3.1. Macrogeometric Analysis

**Figure 9:**
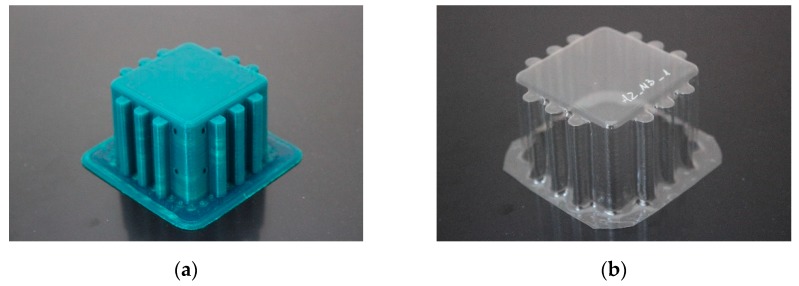
Example images of the results obtained: (**a**) mould A2_M3, (**b**) packaging created with mould A2_M3.

The moulds and the thermoformed packaging had been visually inspected to analyse the final result, Figure 9. Likewise, all the tests carried out had been recorded in order to study the behaviour of the specimens subjected to compression.

Two distances between reliefs had been studied: 3 mm and 6 mm. M1 being the test piece that included 1 relief, M2 and M3 corresponded to 3 reliefs, with a distance of 3 and 6 mm, respectively and M4 and M5 corresponded to the test pieces that had 5 reliefs, 3 and 6 mm, respectively. A2 geometry with 5 reliefs and 6 mm spacing was not evaluated because the size of the specimen face was too small to include these reliefs. Table 2 details the full nomenclature of the designed test specimens.

#### 3.3.2. Mechanical Characterization

**Figure 10:**
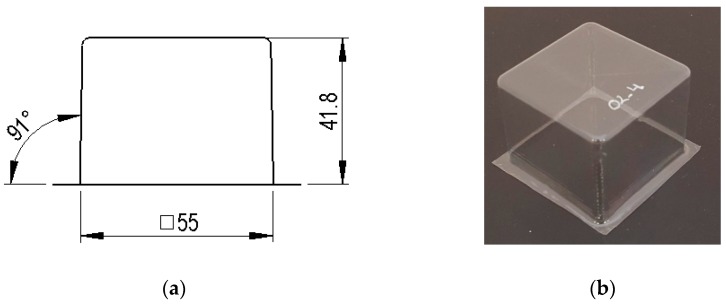
Images and dimensions of the plain test specimen, O2: (**a**) general dimensions; (**b**) test specimen.

A total of 15 tests were performed, including the specimen test without reliefs. The specimens had a square base of dimensions 50 × 50 mm, according to [34]. Figure 10 shows an image with the dimensions of the specimen O2.

The compression tests were carried out according to the guidelines established in Reference [34] and [35]. 4 tests were realized per test specimen typology. 

**Figure 11:**
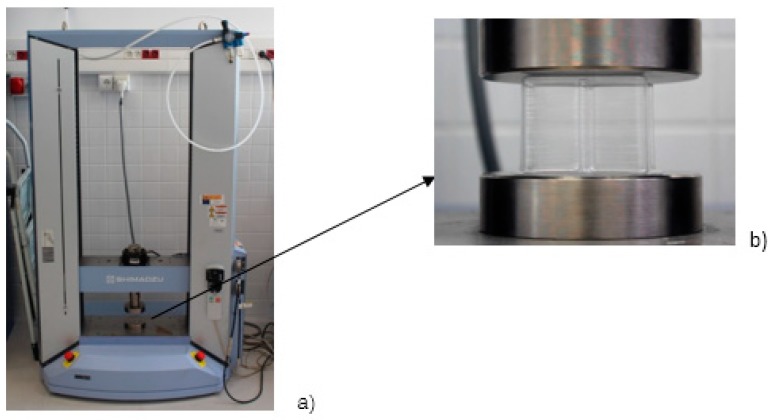
Compression tests: (**a**) general machine view; (**b**) specimen positioning.

The machine used to carry out the mechanical compression tests was the same used for the tensile ones, Figure 11. The load cell was set to 50 KN. As for the tensile tests, the test parameters were handled with Trapezium^®^. The compression speed of the tests was 10 mm/min, according to [35].

The compression tests were recorded using a high-precision digital camera Canon EOS 650D. The recorded images of the compressed specimens were used to relate the real deformation to the measured stress-strain data. This is important, because the recorded axial force, *F_y_*, could be overestimated if folding of the packaging occurs. Also, plastic deformation could be admitted if the packaged product was not affected by it.

The test procedure started with the placement of the specimens on the cylindrical platform of the testing machine, Figure 11b. The load cell approached the specimen and, once the machine had been calibrated, the compression test began. The control parameters set in the program were force in N, time in s and displacement in mm. After carrying out the laboratory tests, a case study was carried out using the same methodology as in the tensile tests, with one of the test pieces used to compare the simulation with the data obtained in the real tests. The simulation, as in the tensile tests, was carried out with Hyperworks Radioss^®^ and Solidworks^®^ where the base was established as fixed and constant speed was included in the upper side.

## 4. Results and Discussion

### 4.1. Tensile Strength

#### 4.1.1. Mechanical Evaluation

Table 3 shows the measured section of geometries studied. A study of the thicknesses on the physical specimens was carried out to obtain the real section, bearing in mind that the material was stretched where the thermoforming process took place. According to this, the specimens presented a different tensile strength given by the type of area and volume generated. 

**Figure 12:**
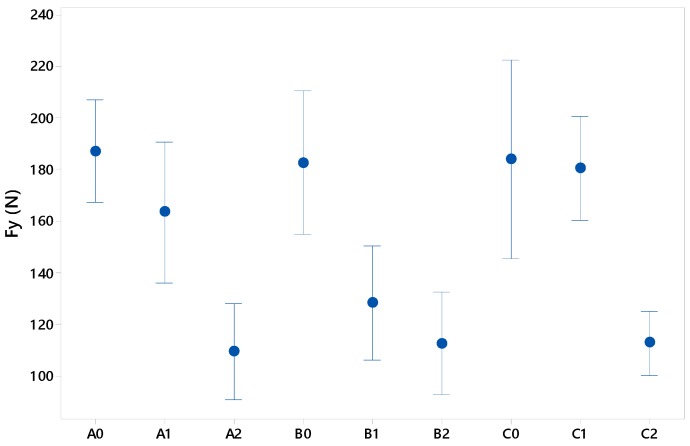
Dispersion of *F_y_* in tensile tests.

As can be seen in Figure 12, geometries A0, B0 and C0 provided higher force values compared to A2, B2 and C2. This is related to the fact that a smaller section provides a greater wall thickness after thermoforming due to the lower stretching suffered by the specimen and generates greater rigidity to the relief.

Indeed, for the smallest packaging, the values reached forces around 185 N, although it is true that it is the A geometry that seems to maintain a lower dispersion in the repetitiveness of the tests. This phenomenon is especially visible in the results for the B0 and C0 reliefs, although it is in the latter geometry where the dispersion of the results is greater, with values of up to 35% lower than the established maximum. This could be due to the fact that the vertices of the square and the triangular geometry act as stress concentration points, which could lead to defects and micro-cracks that lead to premature failure.

Size 1 presented intermediate force values to those compared for size 2 and size 0 with greater variability in the averages obtained. Thus, C geometry presented values close to those reached for the smallest relief with an average of approximately 180 N. However, B geometry obtained values closer to those found in higher relief tests with an average of about 130 N. This seems to reinforce what was previously discussed with respect to the size of the section.

Size 2 presented mean values of about 110 N, about 40% lower than the other sizes. In a deeper analysis of the results, the geometry 2 tests showed values between 90 N and approximately 130 N with smaller dispersions than in previous cases.

Summarizing, the nominal stress analysis shows the trend followed by each A, B and C geometry as a function of their relief. In all three cases there is a tendency for the stress to decrease as the relief increases in size. However, as discussed, the C1 case shows a different trend because its section is proportionally smaller than in cases A1 and B1 and similar to the values obtained for size 0.

#### 4.1.2. Dimensional Evaluation

Due to the fact that the base of the specimen influences the result of the tests, dimensional analysis has been carried out isolating the stress of the relief by means of the dimensional relation given by Equation (5):

(5) $${\sigma y}_{relief}=\left({\sigma y*S}_{rel}\right)/S,$$

where S is the real section of area of the specimen and S*_rel_* is the real section area of the relief.

**Figure 13:**
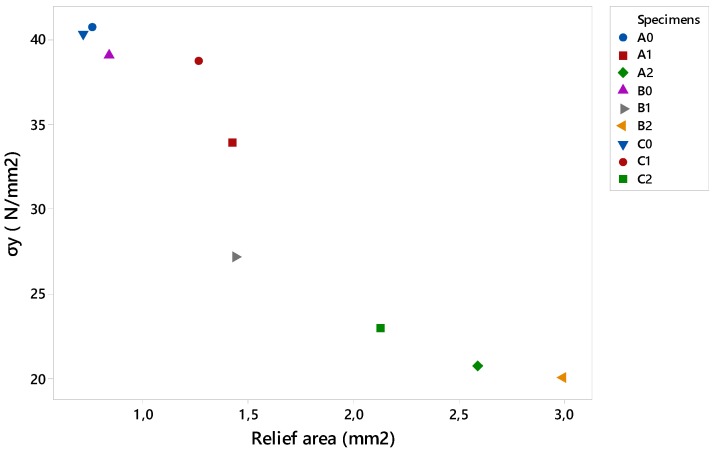
Data of the yield stress with respect to the section of the relief.

Figure 13 details the results obtained according to the middle section, by calculating the stress from the force and area of the relief. It is observed that the geometries A1 and C1, which contain the smaller reliefs, supported a greater tension, especially C1, considering its size. This reinforces the results obtained previously for circular and triangular reliefs with less size. On the other hand, it can also be seen that the three largest geometries bore less stress, as seen in previous sections, thus highlighting the importance of the relationship between the area of the specimen and the relief.

**Figure 14:**
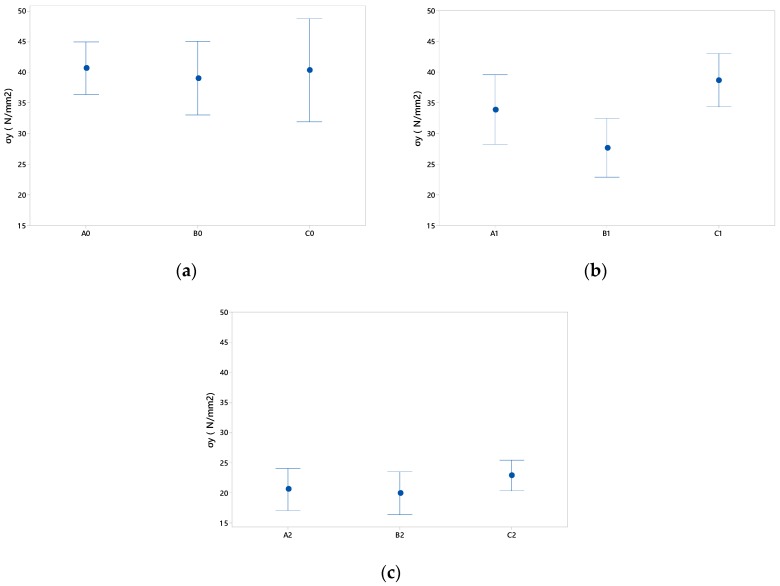
Comparative graph of the average results obtained for the yield stress of the three types of relief (A, B and C): (**a**) for the lower relief measure 0, 1.5 × 1.5 mm; (**b**) for the lower relief measure 1 of 3 × 3 mm; (**c**) for the relief measure 2 of 6 × 6 mm.

To try to have a detailed view of the results by taking into account only the geometry, the dispersion of the results obtained in Figure 14 is shown. 

Regarding the results for size 0, Figure 14a, it is observed that the differences of F_y_ are minimal according to relief and size. This may be due to the fact that the area of the relief with respect to the specimen is minimal and therefore has less influence on the base geometry.

The results of the tests with the specimens with relief size 1, Figure 14b, evidence that there are differences with respect to the dimension of the relief. They also show that the C1 geometry withstands the highest stress and that B1 has the lowest tensile strength. 

Finally, Figure 14c shows the results for size 2. The A2 and B2 reliefs present similar creep stress despite the dimensional differences between them, although the triangular geometry, C2, presents greater strength.

#### 4.1.3. Simulation Validation

**Figure 15:**
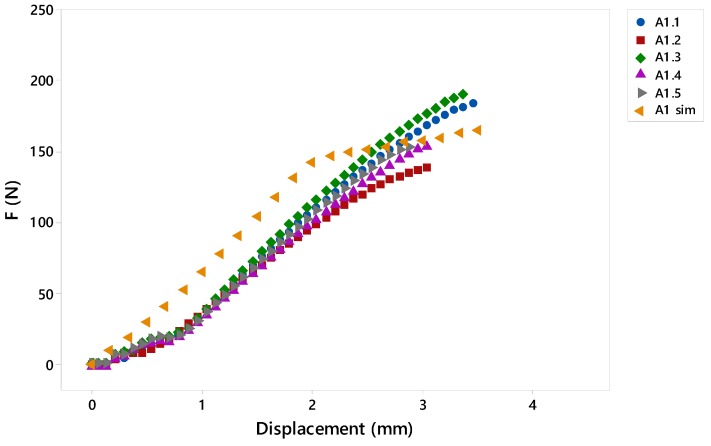
Example comparing the results obtained in the simulation test and the experimental studies.

In general, the curve obtained in the different geometries is partially shifted to the left with respect to the experimental ones, probably due to the placement of the specimen in the testing machine, Figure 15. Another explanation can be given by the flexible characteristic of the specimens where the initial tension varies at the beginning of the test. In this way, it is observed that the specimen began to stress when the jaws move less than 1 mm, that is, there was a delay reflected by the described displacement. 

**Figure 16:**
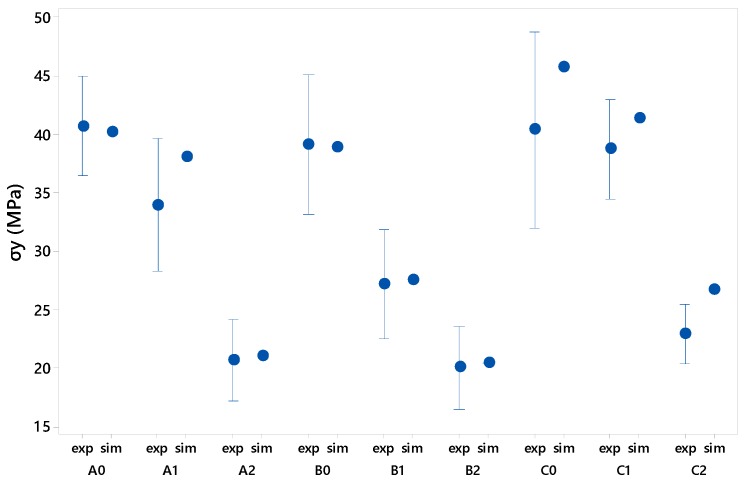
Comparative results of the experimental study (exp) and the simulation carried out (sim) for the yield stress.

On the other hand, the average values of σy obtained experimentally from the five tests and the values obtained in the simulation are detailed in Figure 16. Following [36], it is possible to the values obtained in the simulation with respect to the tests using the following equation:

(6) $$\mathrm{Er}=\frac{\mathrm{Test}\;\mathrm{value}-\mathrm{simulation}\;\mathrm{value}}{\mathrm{test}\;\mathrm{value}}\times 100,$$

where Er is the relative error. 

In view of the results obtained, it can be considered that the simulation model adequately reproduces the mean specimen rupture with a relative error below 2% for circular and square geometries and slightly higher for triangular geometry specimens. This may be due to the concentration of stresses in the main corner of the triangular specimen as a consequence of the scale of the meshing and the simplification of the geometries, this being a singular point of study that shows a distorted behaviour in comparison to the rest of the reliefs. 

The graph in Figure 16 shows that the C0 and C2 specimens, both with triangular geometry, are the ones with the greatest relative error for the reasons discussed above, although it is the C2 type specimen that lies outside the range of dispersion obtained experimentally, being the only set of tests under these conditions. 

It should be noted that for general purposes the simulation results comply to a large extent with the experimental results validating the simulation methodology applied.

### 4.2. Compression Tests

#### 4.2.1. Visual Characterization

As shown in Table 4, in general, for the same size of relief, the film adapts better to the contour of the plastic mould for bigger separation of the reliefs. This is due to the stretching of the film. Thus, the lack of definition that occurs when the reliefs are located at a shorter distance can cause the compressive strength of the container to be reduced.

Similarly, the relief size also affects the type of adaptation of the film on the reliefs. Thus, the larger the relief size, the poorer the definition of the geometry obtained after thermoforming. According to [37] and [38], this is due to the fact that the machine has to exert a greater force to adapt the sheet to the geometry, also producing greater stretching. Thus, in the smaller geometries, A0 and A1, the sheet adapts to the relief, while in the A2 geometry, with a height of 6 mm, there is less definition in the intermediate spaces between reliefs.

It is observed that in the lateral walls the thickness of the sheet was reduced considerably being the part of the sheet where the greatest stretching took place. Therefore, the smaller the distance between reliefs, the less defined the relief geometry.

#### 4.2.2. Mechanical Evaluation

**Figure 17:**
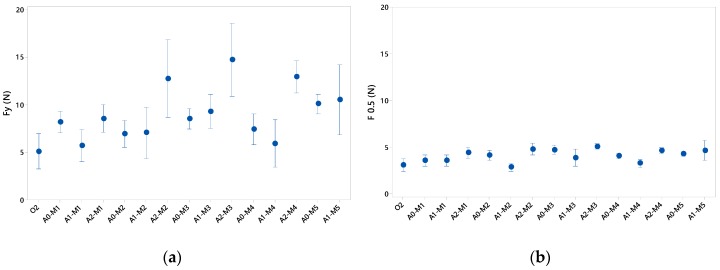
(**a**) Compression forces at stress yield; (**b**) Compression forces for a displacement of 0.5 mm.

The force in the tests made on the thermoformed specimen without reliefs, O2, presented an average F_y_ of 5.13 N, Figure 17a. The standard deviation presented an interval amplitude range of 1.18 N. According to the images collected in the test video, this deviation in the results was due to the fact that the container began to deform on the walls and folds were produced that could result in a different F_y_. Thus, it was observed that, when there was a displacement of 0.5 mm, the container was deformed but no folds were observed. At that moment, the average force collected was 3.10 N and its deviation was 0.43 N, Figure 17b. From the data collected, it can be seen that the 3 sizes, A0, A1 and A2, improved the F-ε properties with respect to the O2 container, although it is true that A2 shows higher values than the rest. Comparing the results obtained by separation and reliefs, the graph shows that slightly higher results are obtained when the reliefs were arranged with a separation of 6 mm (M3 and M5) conserving the same number of reliefs per face: M3 with respect to M2 and M5 with respect to M4. This phenomenon was in good agreement with the videos studied since the greater the relief, the less the deformation of the walls was, due to the fact that a greater distance between reliefs favoured the sheet adaptation to the mould during the thermoforming process.

On the other hand, it should be pointed out that, although A2 has the highest values of force with respect to A0 and A1, it stands out for reflecting higher deviations, especially in A2-M2 and A2-M3, indicating that the behaviour of these containers was more irregular when tested as a consequence of having a higher relief height.

In Figure 17b, it is observed that the recorded force data, for a 0.5 mm of displacement, have greater homogeneity when compared at the same point and not at the yield point, where each test shows different values. Thus, in this case, the results show a similar trend in terms of behaviour based on geometry, relief and separation.

In addition, the dispersion of the data is considerably reduced, which indicates that the tests carried out had a greater homogeneity in the first stages of the test, making possible to clearly distinguish the package that presented better properties. Also, it should be noted that, for deformities greater than 0.5 mm, the packaging could lose its functional properties, thus deteriorating the product contained inside.

#### 4.2.3. Case Study of Validation by FEM

**Figure 18:**
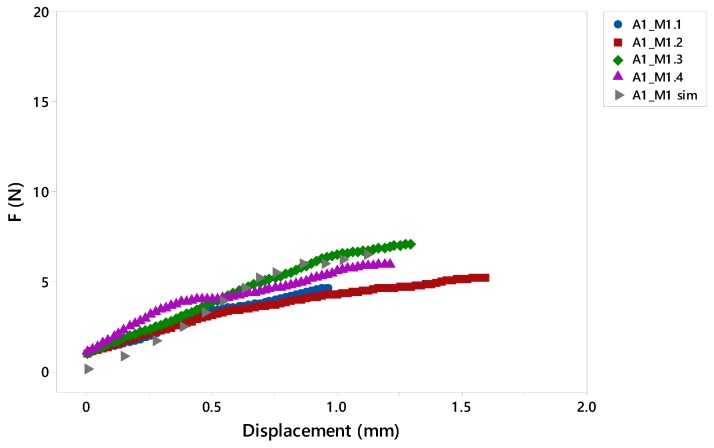
Comparative graph of the results obtained in the compression test by FE.

Figure 18 shows that the compression curve obtained by simulation for the A1-M1 specimen is within the range of the experimentally measured values. When a displacement of 0.5 mm occurred, the obtained force data was also homogeneous with respect to laboratory tests.

Thus, the relative error, according to Equation (6), is 0.14% with respect to the mean data. It is therefore within the dispersion value collected in the performance of the 4 experimental tests.

### 4.3. Relationship between Tensile and Compressive Tests

**Figure 19:**
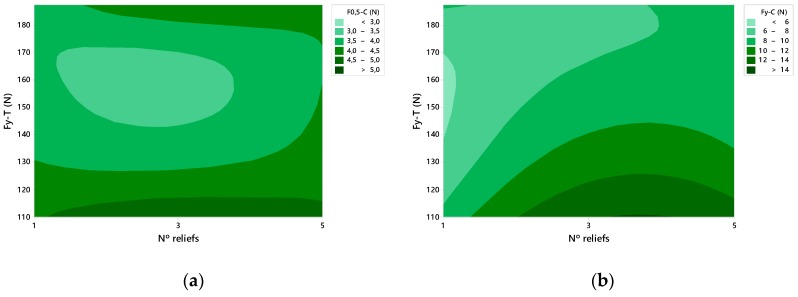
Graph of the compression force, F-C, in relation to the number of reliefs per face and the tensile force at the yield point, F_y_-T, (**a**) at the moment of displacement of 0.5 mm, F0.5-C (**b**) at the yield point, F_y_-C.

Figure 19 shows the area graphs that relate the force data obtained in the tensile tests, F_y_-T, where the reliefs have been studied in a unitary way, with respect to the results of the compressive strength in two stages, F0.5-C and F_y_-C, according to the number of reliefs included per face. Figure 19a relates the results of the F_y_-T with respect to the force at the moment when the displacement has reached 0.5 mm. On the other hand, in Figure 19b the performance is made with respect to the force to compression at the creep point, F_y_-C.

It is observed that the most favourable data of traction and compression, for the force at the moment of 0.5 mm of displacement, were collected with 3 and 5 reliefs per face, Figure 19a. In the case of the F_y_-C, Figure 19b, data reflects that the results that obtain an equilibrium of compressive force between 8 and 10 N in addition to good traction results are the containers presenting 5 reliefs per face.

## 5. Conclusions

The physical tests carried out show that, the higher the relief, the lower the tensile strength compared to smaller sizes. This may be due to the stretching of the sheet on the side walls. However, it provides greater rigidity to the container by favouring compressive strength by introducing relief patterns on the surface of the thermoforming container.

From the geometries studied it can be observed that the semi-circular geometry, A and triangular, C, are the ones that present the best results. Also, the semi-circular geometry, A, adapts better to the shape of the mould since the shape of the relief has no edges.

The analysis carried out shows that the main structural factors are the relationship between the width of the relief and its height. In addition, the type of geometry performed affects the mechanical properties. Likewise, from the comparison of the physical tests and the simulation it is concluded that the correct positioning and tension of the specimen on the testing machine influences the displacement registered in the laboratory tests.

Thus, it can be stated that, by simulation tests, a virtual evaluation method can be established as a basis for the optimization of the design applied to thermoforming packaging. In addition, validation by means of FEM tests gives rise to the possibility of testing new reliefs with more complex geometries. Therefore, it is proposed that, by means of the individualized study of the behaviour of the reliefs, it is possible to compare their mechanical properties. 

Moreover, it was also possible to develop a method that has allowed satisfactory results after the compression tests carried out. The results obtained show that the correct design of the reliefs, according to the number of reliefs used and their disposition, can favour the improvement of the mechanical properties of the containers. Specifically, the reliefs with greater distance increase the resistance of the packaging.

In comparison with the conventional design process of thermoforming packaging, this new procedure, aimed at the study of reliefs using low-cost moulds of AM, facilitates the realization of more innovative and efficient designs through the study of geometries, number of reliefs and their positioning. In this sense, it is possible to design containers with lower material costs by improving the mechanical properties using materials of reduced thickness and increasing the protection of the food.

Along these lines, a field of study was opened on the inclusion of complex relief patterns on thermoformed surfaces. The realization of complex patterns can be possible by means of the application of FDM moulds. Also, these moulds could be used to make cut series and thus improve personalization and brand identity. In short, this work presents a tool for designers that facilitates customization, eco-design and allows them to optimize their mechanical properties. 

## Figures and Tables

**Table 1:** Properties of PET laminate.

Thickness	Surface Treatment	Tensile Strength	Ductility	Impact Resistance	Density
0.180 mm ± 3%	Silicone on both outer sides	>50 N/mm^2^	220%	>3 KJ/m^2^	1.33 gr/cm^2^

**Table 2:** Nomenclature of reliefs according to the type of relief, distance and dimensional correspondence.

Relief	Specimen	Nº Reliefs	Relief Dimensional Proportion	Relief Size (mm)	Distance between Reliefs (mm)
-	O2	0	-	-	-
A0	M1	1	a/2 × a/2	1.5 × 1.5	-
A0	M2	3	a/2 × a/2	1.5 ×1.5	3
A0	M3	3	a/2 × a/2	1.5 × 1.5	6
A0	M4	5	a/2 × a/2	1.5 × 1.5	3
A0	M5	5	a/2 × a/2	1.5 × 1.5	6
A1	M1	1	a × a	3 × 3	-
A1	M2	3	a × a	3 × 3	3
A1	M3	3	a × a	3 × 3	6
A1	M4	5	a × a	3 × 3	3
A1	M5	5	a × a	3 × 3	6
A2	M1	1	2 a × 2a	6 × 6	-
A2	M2	3	2 a × 2a	6 × 6	3
A2	M3	3	2 a × 2a	6 × 6	6
A2	M4	5	2 a × 2a	6 × 6	3

**Table 3:** Results of the area of specimens and tensile strength.

Relief	Measured S (mm^2^)	F_y_ (N)	Standard Deviation (N)
**A0**	4.60	187.24	15.87
**A1**	4.82	163.49	22.04
**A2**	5.31	109.75	15.02
**B0**	4.68	182.78	22.56
**B1**	4.73	128.53	17.74
**B2**	5.63	112.71	16.07
**C0**	4.56	184.06	30.90
**C1**	4.66	180.38	16.14
**C2**	4.93	112.84	10.09

**Table 4:** Kinds of specimens made according to the number of reliefs and disposition.

	M1	M2	M3	M4	M5
A0	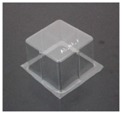	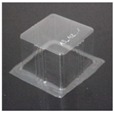	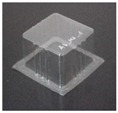	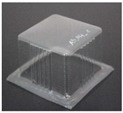	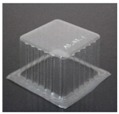
A1	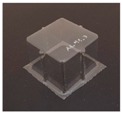	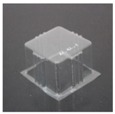	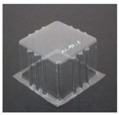	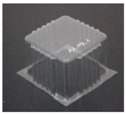	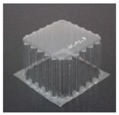
A2